# The eIF4F and eIFiso4F Complexes of Plants: An Evolutionary Perspective

**DOI:** 10.1155/2012/287814

**Published:** 2012-05-07

**Authors:** Ryan M. Patrick, Karen S. Browning

**Affiliations:** Department of Chemistry and Biochemistry and the Institute for Cell and Molecular Biology, The University of Texas at Austin, Austin, TX 78712, USA

## Abstract

Translation initiation in eukaryotes requires a number of initiation factors to recruit the assembled ribosome to mRNA. The eIF4F complex plays a key role in initiation and is a common target point for regulation of protein synthesis. Most work on the translation machinery of plants to date has focused on flowering plants, which have both the eIF4F complex (eIF4E and eIF4G) as well as the plant-specific eIFiso4F complex (eIFiso4E and eIFiso4G). The increasing availability of plant genome sequence data has made it possible to trace the evolutionary history of these two complexes in plants, leading to several interesting discoveries. eIFiso4G is conserved throughout plants, while eIFiso4E only appears with the evolution of flowering plants. The eIF4G N-terminus, which has been difficult to annotate, appears to be well conserved throughout the plant lineage and contains two motifs of unknown function. Comparison of eIFiso4G and eIF4G sequence data suggests conserved features unique to eIFiso4G and eIF4G proteins. These findings have answered some questions about the evolutionary history of the two eIF4F complexes of plants, while raising new ones.

## 1. Introduction

In eukaryotes, posttranscriptional gene regulation at the level of translation initiation is an important mechanism [1]. The process of translation initiation begins with the eIF4F complex, made up of the subunits eIF4E, which recognizes the 7-methylguanosine (m^7^G) cap on the 5′ end of mRNA, and eIF4G, which binds to eIF4E and serves as a scaffold for other initiation factors [2]. eIF4G has sites for binding poly(A)-binding proteins (PABPs), which bind to the poly(A)-tail at the 3′ end of the mRNA, effectively allowing the eIF4F complex to circularize the mRNA molecule [3]. eIF4G also has RNA binding activity which may promote association with mRNA and improve eIF4E cap recognition [4]. eIF4G additionally binds the RNA helicase eIF4A [5], which promotes ATP-dependent unwinding of RNA secondary structure in a manner promoted by eIF4G and eIF4B [6]. The 43S preinitiation complex, made up of the 40S ribosomal subunit, eIF2 bound to GTP and Met-tRNA^Met^_*i*_, eIF3, eIF1, eIF1a, and eIF5 [2], is recruited to the mRNA by eIF4G through contacts with eIF3 [7] as well as eIF5 and eIF1 [8]. The docking of the 43S preinitiation complex is followed by scanning for the AUG start codon and joining of the 60S ribosomal subunit to begin translation [2]. The placement of the eIF4F complex at the beginning of this process makes it a key point for regulation of protein synthesis [9].

 Flowering plants have two distinct isoforms of the eIF4F complex. In addition to the evolutionarily conserved eIF4F complex made up of eIF4E and eIF4G, they also have a plant-specific eIFiso4F complex made up of eIFiso4E and eIFiso4G [10, 11]. Wheat eIF4F and eIFiso4F have been shown to have differential effects on translation of various RNAs [12]. It has been reported that eIF4E-binding to eIF4G is very tight (0.18 nM K_D_) and eIFiso4E-binding to eIFiso4G is similarly tight (0.08 nM K_D_), while mixed complexes of eIF4E to eIFiso4G and eIFiso4E to eIF4G have ~80–100-fold less tight binding than their preferred partner; however, the mixed complexes retain activity *in vitro *[13]. *Arabidopsis thaliana *mutant plants with only a mixed complex of eIFiso4G and eIF4E are able to survive; but, those plants with only eIF4G and eIFiso4E do not appear to be able to progress through a normal developmental program (Mayberry and Browning, unpublished observations). These results suggest that unique properties are associated with the two cap-binding complexes and their subunits in plants.

The increasing amount of sequence data from Viridiplantae (the monophyletic group of green plants, including the green algae and land plants) has made it possible to ask questions about the evolutionary history of the eIF4F and eIFiso4F complexes. Essentially all work to date on the translation machinery of Viridiplantae has been done in flowering plants. This work seeks to clarify the distribution of eIF4F and eIFiso4F subunit genes through Viridiplantae and identifying sequence traits in order to better understand the evolutionary significance of these complexes.

## 2. Materials and Methods

Plant eIF4F/eIFiso4F subunit protein sequences were obtained by BLAST of genome databases including NCBI [14], Joint Genome Institute [15], Phytozome [16], Sol Genomics Network [17], the Strawberry Genome [18], and Cacao Genome Database (http://www.cacaogenomedb.org/). Upstream genomic regions were translated using the ExPASy Translate tool [19] and were in some cases used where annotated eIF4G protein sequences may be incomplete. eIF4G and eIFiso4G alignments were performed by ClustalW2 [20] with manual adjustments (see Supplementary Table 1 in Supplementary Material available online at doi:10.1155/2012/287814 for a list of genes/loci used). eIF4E and eIFiso4E alignment and phylogeny were generated by MAFFT [21].

## 3. Results and Discussion

### 3.1. eIFiso4E Appears in Flowering Plants

**Figure 1:**
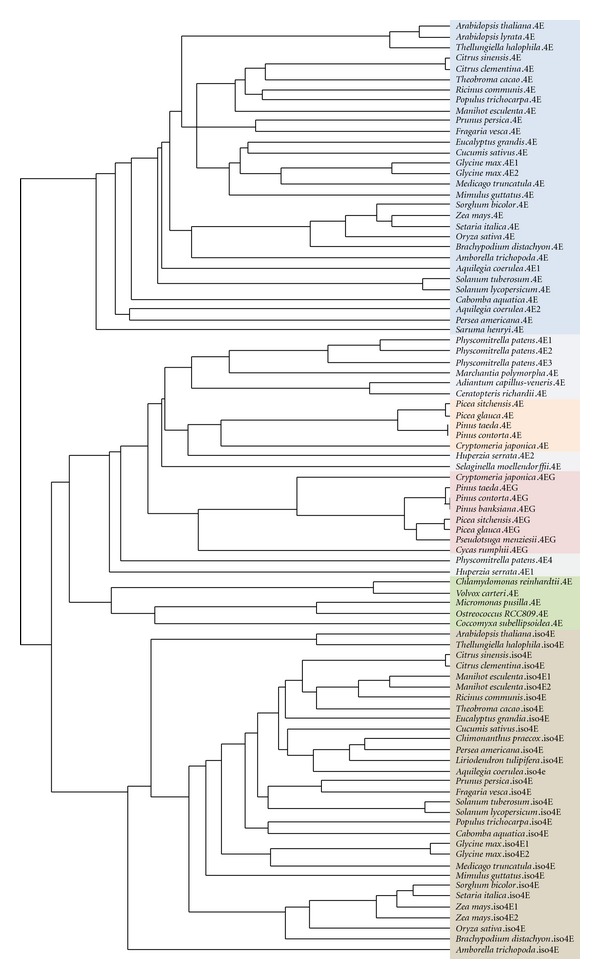
Phylogeny of Viridiplantae eIF4E and eIFiso4E. eIF4E_gs_ genes of gymnosperms are labeled eIF4EG. Phylogeny generated by alignment of eIF4E, eIFiso4E, and eIF4E_gs_ genes using MAFFT version 6 [21].

All flowering plants with available completed genome sequences encode eIF4E and eIFiso4E proteins (Table 1). Most Viridiplantae also encode the conserved additional eIF4E family member 4EHP (also known as nCBP in plants) [22], though it is lost in green algae. Additionally, some plants, like *A. thaliana*, encode eIF4E-like genes with divergence from the canonical plant eIF4E sequence which we term eIF4E1b genes (Patrick and Browning, manuscript in preparation). To address the lineage of eIF4E and eIFiso4E, a phylogeny of eIF4E genes from Viridiplantae was constructed (Figure 1).

To our knowledge, it has not been previously noted that eIFiso4E first appears at the emergence of flowering plants; it is not present in the genomes of the bryophyte *Physcomitrella patens*, the lycophyte *Selaginella moellendorffii*, or green algae, and there is no expressed sequence tag (EST) support for eIFiso4E before angiosperms evolved. *Amborella trichopoda*, the earliest diverging angiosperm known [23], has EST support for both eIF4E and an early eIFiso4E, and ESTs from other early angiosperms (such as the aquatic flowering plant *Cabomba aquatica*, see Figure 1) support a fully developed flowering plant eIFiso4E.

We have also found that gymnosperms have two forms of eIF4E, with one resembling the more conserved plant eIF4E and one being a divergent form of eIF4E that is distinct from eIFiso4E, which we term eIF4E_gs_ (eIF4E Gymnosperm). There is currently good EST support for eIF4E_gs_ within conifers, as well as evidence of its presence in the cycad *Cycas rumphii* (Figure 1). Research of the translation machinery in conifers would be needed to address whether eIF4E_gs_ has a preferred binding partner in eIFiso4G or eIF4G, creating a parallel form of eIFiso4F in gymnosperms. It is unclear whether gene duplication happened in the common ancestor of gymnosperms and angiosperms, with the duplicated eIF4E diverging to eIF4E_gs_ in gymnosperms and to eIFiso4E in angiosperms, or whether parallel gene duplication and divergence happened in each lineage; it is interesting in either case that the development of a second distinct eIF4E in plants seems coincident with transition to seed-based reproduction.

### 3.2. Distribution of eIF4G and eIFiso4G in Viridiplantae

**Figure 2:**
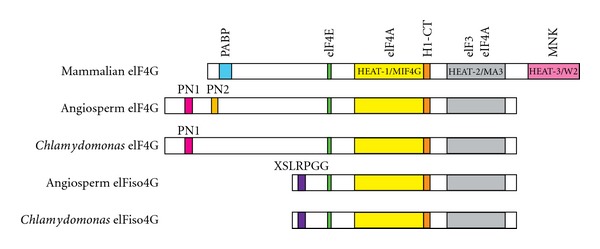
Domain organization of eIF4G and eIFiso4G from mammals, angiosperms, and the green algae *Chlamydomonas reinhardtii*. Plant eIF4G and eIFiso4G share the core organization of the eIF4E-binding site, HEAT-1/MIF4G domain, H1-CT motif, and HEAT-2/MA3 domain with mammals, but do not have the C-terminal HEAT-3 domain. The eIF3- and eIF4A-binding regions are thought to be maintained between all shown isoforms. Plant eIF4G has a longer N-terminus than mammals and contains the plant-specific 4G-PN1 and 4G-PN2 motifs as shown. *Chlamydomonas *eIF4G has a 4G-PN1-like sequence but no 4G-PN2 motif, while other green algae may have a 4G-PN2 motif but no 4G-PN1 motif. eIFiso4G is remarkably well conserved across plants, with the N-terminal XSLRPGG motif maintained from green algae to angiosperms.

 The domain structure of eIF4G in plants is organized similarly to mammals, with a shared core structure of an eIF4E-binding site, the HEAT-1/MIF4G and HEAT-2/MA3 domains which bind eIF4A and contribute to mRNA scanning [24], and a long N-terminus with little identified structure [25]. Plant eIF4G differs from mammalian eIF4G in that it lacks the C-terminal HEAT-3/W2 domain. Plant eIFiso4G is similar in structure to eIF4G, but lacks the long N-terminus (see Figure 2).

 One of the most interesting questions regarding the translation machinery of plants is why they contain both eIF4G and the plant-specific isoform eIFiso4G. In flowering plants, these proteins form distinct eIF4F (eIF4G with eIF4E) and eIFiso4F (eIFiso4G with eIFiso4E) complexes, that differ in their ability to promote translation of structured mRNAs *in vitro *[26]. Plant viruses often require one of these complexes for replication, but not the other, and the genes for the subunits of eIF4F or eIFiso4F have been identified as virus resistance genes for many types of plant viruses [27]. Most flowering plants with completed genomes available have more than one eIFiso4G gene (Table 1); *A. thaliana* has two, with the eIFiso4G1 gene being more highly expressed than eIFiso4G2. They appear to have overlapping functions, since deletion of either eIFiso4G subunit has little effect, but simultaneous deletion leads to a severe phenotype [28].

 Flowering plants with completed genomes are about evenly divided between those that have a single copy of eIF4G and those that have two or more, but it is more common for the eIFiso4G copy number to be higher than eIF4G than vice versa (Table 1). *A. thaliana* has one eIF4G gene, and interestingly deletion of eIF4G has little effect (Mayberry and Browning, unpublished observations), in contrast to the severe growth phenotype of the eIFiso4G double mutant [28]. Nearly all Viridiplantae species which currently have sequenced genomes available contain genes for both eIF4G and eIFiso4G (*Chlorella variabilis* is a possible exception, as it appears to encode only eIFiso4G). This evolutionary conservation suggests that, while the genes have overlapping functions in translation initiation, each may have important specific roles in gene regulation as well.

 As there was no eIFiso4E present before the evolution of angiosperms, it is unclear whether the binding partner of eIFiso4G at the conserved 4E-binding site (see below) was eIF4E or 4EHP in earlier Viridiplantae. Wheat eIFiso4G can form a complex with 4EHP that has some capacity to enhance translation initiation [22]; however, in *A. thaliana,* 4EHP does not appear to form a complex with eIF4G (Patrick and Browning, unpublished observations). 4EHP does not appear to be present in green algae (Table 1), leaving eIF4E the most likely option to form a complex with eIFiso4G in that lineage. As the function of eIFiso4G has only been studied in flowering plants that express eIFiso4E and form the eIFiso4F complex, research would be necessary to confirm that eIFiso4G has similar roles in translation initiation in nonflowering plants.

### 3.3. The N-Terminus of Plant eIF4G

**Figure 3:**
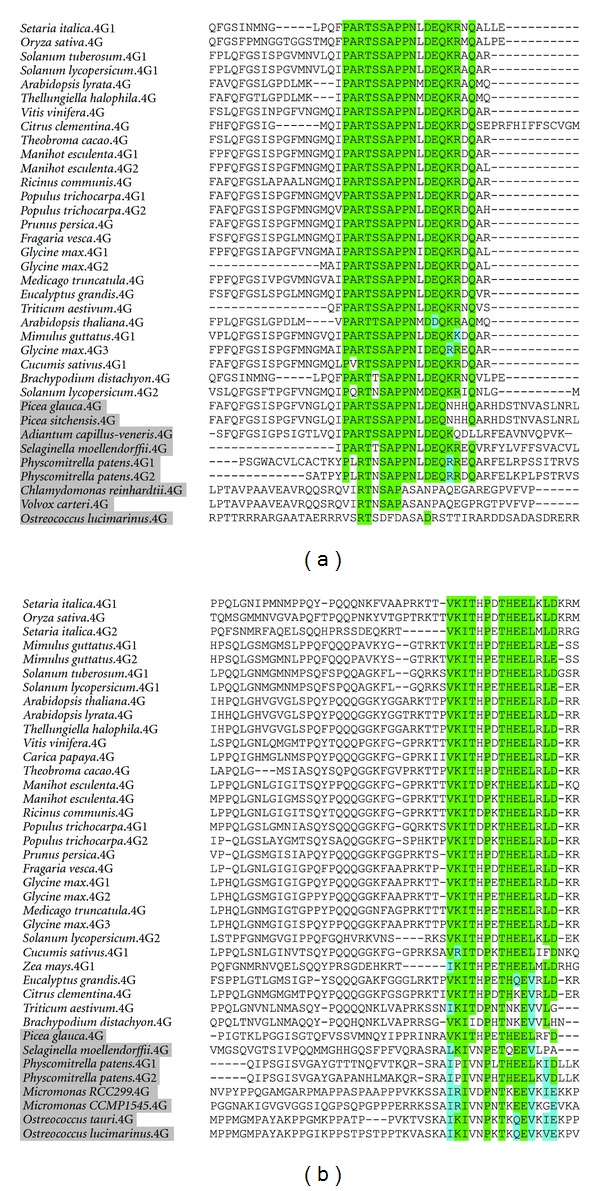
The N-terminal motifs of eIF4G. Residues highlighted in green have identity to the consensus sequence, and residues highlighted in blue have similarity. Genes of nonflowering plants and green algae are shaded grey. (a) The PG-N1 motif with consensus sequence PARTSAPPNxDEQKRxQ. (b) The PGN-2 motif with consensus sequence VKITxPxTHEELxLD.

Due to poor sequence conservation in the N-terminus, there is often difficulty annotating the eIF4G start site, especially outside of angiosperms. Based on available genomic information from flowering plants, we have been able to identify two conserved motifs in the N-terminal region, referred to here as the 4G-PN1 and 4G-PN2 sites (plant eIF4G N-terminal motif 1 and 2). 4G-PN1 is 17 amino acids long, with the consensus sequence PARTSAPPNxDEQKRxQ (Figure 3(a)), and appears 180 amino acids into *A. thaliana* eIF4G. 4G-PN2 is 15 amino acids long, with the consensus sequence VKITxPxTHEELxLD (Figure 3(b)), and appears 375 amino acids into the *A. thaliana* eIF4G. The region N-terminal of 4G-PN1 and between 4G-PN1 and 4G-PN2 is poorly conserved at a sequence level in plants but the positions of the two motifs and length of the intervening sequence are maintained. The 4G-PN2 motif is followed by a long poorly conserved region leading into the 4E-binding site and HEAT-1 domain. The role of these motifs, whether structural or supporting protein-protein interactions, is not known.

 Though the 4G-PN1 and 4G-PN2 motifs are present upstream of the eIF4G HEAT-1 in almost all available Viridiplantae genome sequences, they are sometimes not included in the predicted protein coding sequence. They are present in the genome of *P. patens* and *S. moellendorffii*, as well as EST evidence supporting their existence in the conifer *Picea glauca*, which supports a conserved long N-terminus for eIF4G at least back to the emergence of land plants. Further investigation will be needed to determine if there are alternative splicing and translation initiation sites giving rise to multiple forms of eIF4G in plants. Supporting proteomic data is needed as well to fully understand the role of these motifs.

### 3.4. eIF4G of Green Algae

 Green algae genomes currently annotate eIF4G as several different lengths, with *Chlamydomonas reinhardtii* being predicted as the same length as vascular plant eIF4G, but the close relative *Volvox carteri* being annotated without the N-terminus though its sequence is present in the genome. These green algae encode a 4G-PN1-like motif at the proper location (Figure 3(a)), but do not appear to have a PN2-like motif. *Ostreococcus* and *Micromonas* species have their eIF4G annotated as severely truncated, to the point where the 4E-binding sequence is not included, though it is encoded in the genome. Assuming the annotations are erroneously short, a 4G-PN2-like motif is encoded at the proper location upstream of the eIF4G HEAT-1 domain (Figure 3(b)); however, no 4G-PN1-like motif can be found.

 These lines of evidence support the possibility of a common Viridiplantae ancentral eIF4G with a full length N-terminus containing the 4G-PN1 and 4G-PN2 motifs. If this is the case, either motif may have been lost in some algae lineages, while both were maintained in the land plant lineage.

### 3.5. The H1-CT Site in Plants

**Figure 4:**
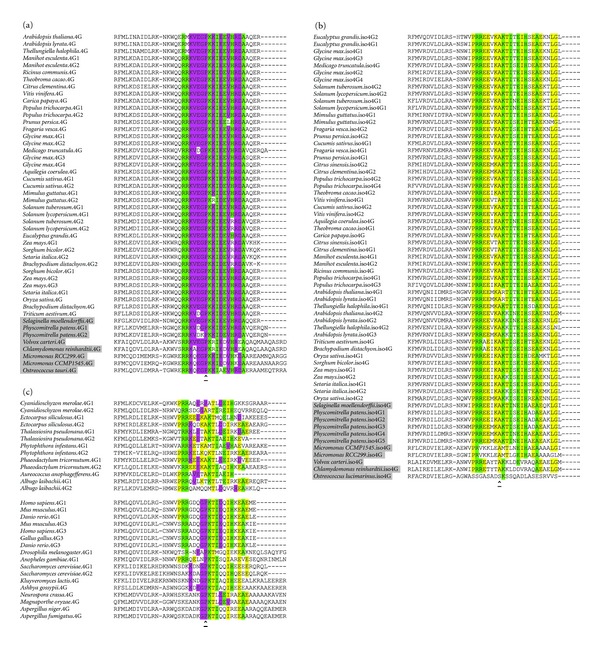
The H1-CT motif of eIF4G and eIFiso4G. Residues highlighted in green have identity to the shared core sequence RRx_5_KxIxExHxxA. The arrow identifies the site of the *cum2* mutation in eIF4G. (a) The H1-CT motif of eIF4G. Residues highlighted in purple have identity to the unique residues of the eIF4G H1-CT motif RRVEGPKKI(D/E)EVHRDA. Genes of nonflowering plants and green algae are shaded grey. (b) The H1-CT motif of eIFiso4G. Residues highlighted in yellow have identity to the unique residues of the eIFiso4G H1-CT motif PRREExKAKTIxEHxEAExxLG. Genes of nonflowering plants and green algae are shaded grey. (c) The H1-CT motif of eIF4G genes of heterokonts, animals, and fungi. Residues are highlighted according to their identity to the shared core motif (green), the motif of plant eIF4G (purple), or the motif of eIFiso4G (yellow).

 The *cum2* mutation in *A. thaliana* was identified as a point mutation of a proline residue in eIF4G that inhibits replication of *Cucumber mosaic virus* [29]. Interestingly, this mutation occurs at a motif that is well conserved in eukaryotes, with the proline at this location conserved in animals and fungi. The motif, found between the end of the HEAT-1 domain and the predicted eIF3 binding site, has previously been identified as the H1-CT motif [25], conserved in fungi and animals, and here we provide evidence that this motif is conserved in most eukaryotic eIF4G proteins (Figure 4).

 The core shared motif of the H1-CT region in plant eIF4G and eIFiso4G, which is also well conserved in other eukaryotes, is RRx_5_KxIxExHxxA (Figure 4). The residues around this core are divergent in eIF4G and eIFiso4G, the eIF4G motif at the site being RRVEGPKKI(D/E)EVHRDA (Figure 4(a)) and for eIFiso4G being PRREExKAKTIxEHxEAExxLG (Figure 4(b)). The H1-CT motif in mammals and yeast shares similarities with both the eIF4G and eIFiso4G motifs (Figure 4(c)). The reason for the difference at this motif in the two plant isoforms is not clear, but it is useful for differentiation between divergent eIF4G and eIFiso4G genes.

### 3.6. Is the Origin of eIFiso4G Outside Viridiplantae?

**Figure 5:**
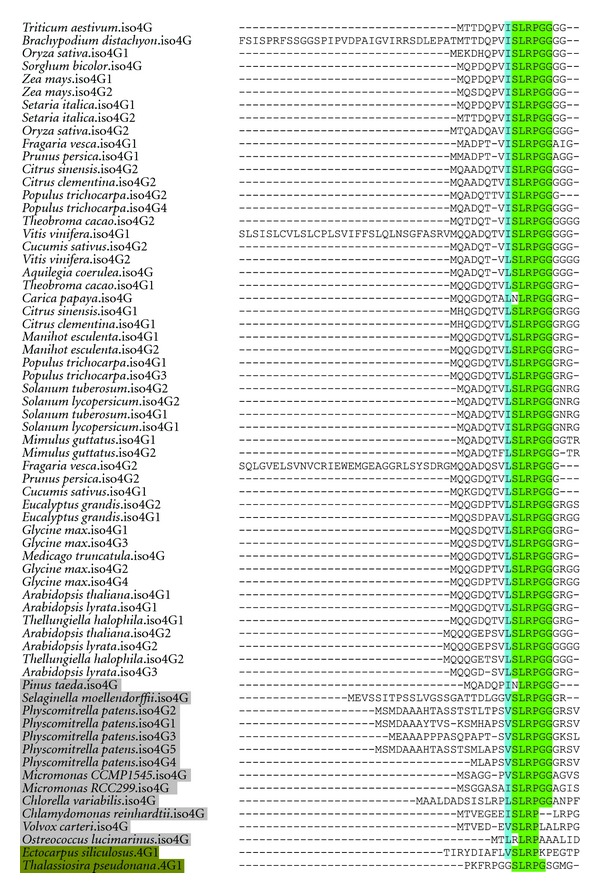
The N-terminal XSLRPGG motif of eIFiso4G. Residues highlighted in green have identity to the consensus sequence, and the variable hydrophobic residue is highlighted in blue. Genes of nonflowering plants and green algae are shaded grey. Genes of the heterokont eIF4G sequences containing this motif are shaded in brown.

 A second site useful for identification of eIFiso4G genes is a conserved N-terminal sequence of XSLRPGG (Figure 5), with X being a hydrophobic amino acid (I, V, or L). This sequence is conserved in eIFiso4G throughout the Viridiplantae lineage, but is not present in eIF4G. The purpose of this conserved motif is unknown, as N-terminal truncations of eIFiso4G lacking this sequence were found to bind eIFiso4E, eIF4A, synthesize polypeptides, and hydrolyze ATP at wild-type levels [30].

 While eIFiso4G is present in all Viridiplantae, it is not clear whether the origin of the plant-specific isoform of eIF4G was before or after the divergence of Viridiplantae. Interestingly, two heterokonts, the brown algae *Ectocarpus siliculosus* and the marine diatom *Thalassiosira pseudonana,* encode a sequence similar to the eIFiso4G XSLRPGG motif at the correct position upstream of an eIF4G HEAT-1 domain. The *E. siliculosus* gene also bears more similarity to eIFiso4G than eIF4G at the H1-CT motif, while the *T. pseudonana* has similarities to both (Figure 4(c)). The red algae *Cyanidioschyzon merolae*, more closely related to Viridiplantae [31], encodes two eIF4G genes, but they are divergent to the point it is not possible to identify them as either eIF4G or eIFiso4G homologs. The *E. siliculosus* gene may contribute evidence of a conserved eIFiso4G outside of Viridiplantae, but there is not enough support at this time to definitively state that the origin of eIFiso4G predates Viridiplantae.

### 3.7. The 4E-Binding Site of eIF4G and eIFiso4G

**Figure 6:**
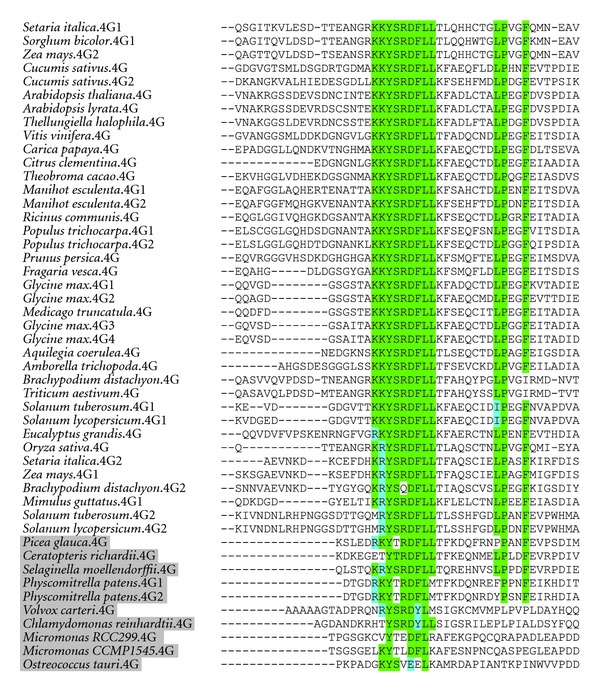
The 4E-binding site of plant eIF4G. Residues highlighted in green have identity to the consensus sequence KKYSRDFLLx_8_LPxxF, and residues highlighted blue have similarity. Genes of nonflowering plants and green algae are shaded grey.

**Figure 7:**
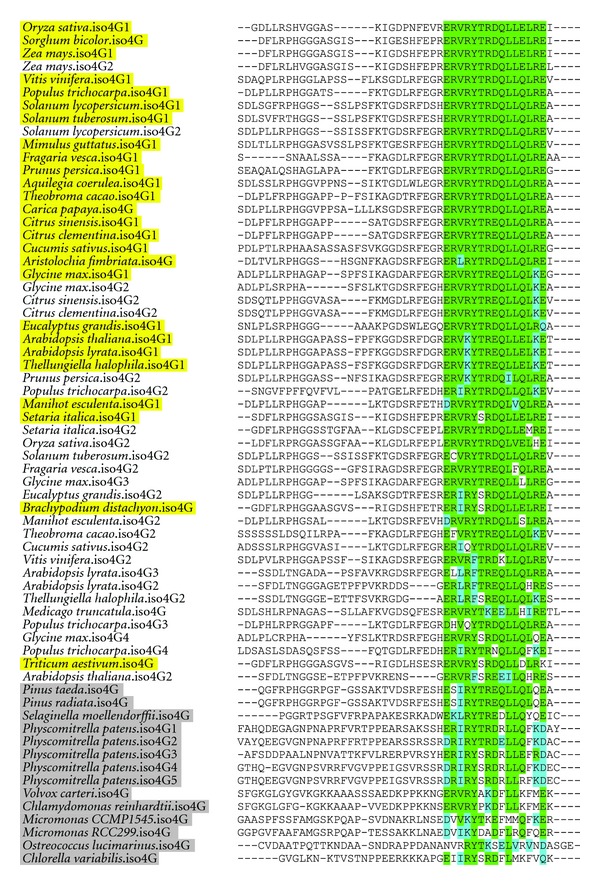
The 4E-binding site of plant eIFiso4G. Residues highlighted in green have identity to the consensus sequence ERVRYTR(D/E)QLLZLRE, and residues highlighted blue have similarity. Genes of nonflowering plants and green algae are shaded grey. Plants generally have one copy of eIFiso4G that closely resembles the consensus sequence; this primary copy is highlighted in yellow. Secondary copies, which are unhighlighted, may diverge from this sequence.

As eIF4G and eIFiso4G prefer to form discrete complexes with eIF4E and eIFiso4E, respectively [6], we used alignment of known sequences for angiosperm eIF4G and eIFiso4G to find if they have distinct 4E-binding motifs and whether the 4E-binding site in these proteins changed after the evolution of eIFiso4E. eIF4G has a well-conserved 4E-binding site sequence of KKYSRDFLLx_8_LPxxF, which appears in its flowering plant form as early as the lycophyte *S. moellendorffii* (Figure 6). The eIFiso4G site for 4E binding is ERVRYTR(D/E)QLLZLRE (Z being Glu or Gln) (Figure 7). Interestingly, it seems common for plants to have one eIFiso4G copy closely matching this consensus sequence, while other copies may diverge from this sequence. For example, *A. thaliana* eIFiso4G1 is close to the consensus sequence, while eIFiso4G2 diverges at several residues. eIFiso4G2 copurifies with eIFiso4E and has similar activity to eIFiso4G1 *in vitro *[12], so it is unclear at this time whether these differences are meaningful.

The flowering plant 4E-binding sequence of eIFiso4G seems nearly fully formed in the bryophyte *P. patens*, and the sequence in green algae eIFiso4G is roughly as similar to its angiosperm counterpart as the green algae eIF4G 4E-binding site is to its angiosperm version. One might expect the 4E-binding sites to have evolved after the emergence of eIFiso4E to each bind their preferred partner and discriminate against the other, but it seems in both cases the 4E-binding site was well formed before eIFiso4E evolved and has changed little since. The discrimination may therefore be at a site on the large subunit away from the identified 4E-binding site, or it may have evolved on the 4G-binding interface of eIF4E and eIFiso4E.

## 4. Conclusions

 The increasing availability of genomic sequences from Viridiplantae has helped clarify the evolutionary history of the flowering plant eIF4F and eIFiso4F complexes, but has also raised many new questions. The discovery that evolution of eIFiso4G occurred long before eIFiso4E is surprising; *in vitro* observations on the eIFiso4F complex of wheat [13–26] and* Arabidopsis* [12] as well as the ability for either eIFiso4E or eIFiso4G gene disruptions to confer resistance to *Lettuce mosaic virus*, *Plum pox virus*, and *Turnip mosaic virus *in *A. thaliana *[32] point to a strongly intertwined role for eIFiso4E and eIFiso4G. This opens up several questions. Before the evolution of eIFiso4E, was eIF4E shared between eIF4G and eIFiso4G, or was 4EHP involved? Does eIFiso4G promote translation in green algae and early land plants, as it seems to in flowering plants, or did it have a different role altogether? What is the relationship between the evolution of flowering plants and the coincident appearance of eIFiso4E, which appears conserved in all available angiosperm sequences? Future work will hopefully begin to answer these questions and should build toward an understanding of the function in flowering plants of the eIF4F and eIFiso4F complexes.

 While mutational and deletion studies have been performed on eIFiso4G [30, 33], less analysis has been published on the activity of different domains of plant eIF4G, and the role of the N-terminal region remains mysterious. Deletion of a significant portion of the eIF4G N-terminus has little effect *in vitro* on translational activity ([34] and Mayberry and Browning, unpublished observations) suggesting the N-terminus may have a regulatory or unknown function. The identification of two N-terminal motifs in the plant eIF4G conserved back to at least the evolution of land plants and possibly as far back as the root of Viridiplantae implies that the N-terminal region does have some important function. Future studies will be necessary to determine whether these motifs are involved in interactions with other proteins (possibly to PABP, the binding site of which has not been identified in plant eIF4G) and to discover whether the N-terminus contributes to translation initiation or to some other as yet unrecognized function(s) of eIF4G.

## Supplementary Material

eIF4G and eIFiso4G gene names used for alignments in this study. Gene ID refers to identifiers used for a gene locus or transcript in the NCBI database or the genome project for that organism.

## Figures and Tables

**Table 1:** Distribution of eIF4F subunit genes in Viridiplantae. Nonflowering plants and green algae are bold.

	eIF4G	eIFiso4G	eIF4E	eIFiso4E	4EHP	eIF4E1b
*Arabidopsis thaliana*	1	2	1	1	1	2
*Arabidopsis lyrata*	1	3	1	1	1	2
*Thellungiella halophila*	1	2	1	1	1	1
*Carica papaya*	1	1	1	1	1	0
*Theobroma cacao*	1	2	1	1	1	0
*Citrus clementina*	1	2	1	1	1	0
*Citrus sinensis*	1	2	1	1	1	0
*Eucalyptus grandis*	1	2	1	1	1	1
*Solanum tuberosum*	2	2	1	1	1	0
*Prunus persica*	1	2	1	1	1	0
*Fragaria vesca*	1	2	1	1	1	1
*Cucumis sativus*	2	2	1	1	1	0
*Glycine max*	4	4	2	2	2	0
*Medicago truncatula*	1	1	1	1	1	0
*Populus trichocarpa*	2	4	1	2	1	0
*Ricinus communis*	1	1	1	1	1	0
*Manihot esculenta*	2	2	1	2	2	0
*Vitis vinifera*	1	2	1	1	1	0
*Mimulus guttatus*	2	2	1	2	1	0
*Aquilegia coerulea*	1	2	2	1	1	0
*Sorghum bicolor*	2	1	1	1	1	0
*Zea mays*	3	2	2	2	1	0
*Setaria italica*	2	2	1	1	1	0
*Oryza sativa*	1	2	1	1	1	0
*Brachypodium distachyon*	2	1	1	1	1	0
**Selaginella moellendorffii**	**2**	**2**	**4**	**0**	**1**	**0**
**Physcomitrella patens**	**2**	**5**	**4**	**0**	**1**	**0**
**Chlamydomonas reinhardtii**	**1**	**1**	**1**	**0**	**0**	**0**
**Volvox carteri**	**1**	**1**	**1**	**0**	**0**	**0**
**Micromonas pusilla**	**1**	**1**	**1**	**0**	**0**	**0**

## References

[1] Merrick WC (2010). Eukaryotic protein synthesis: still a mystery. *Journal of Biological Chemistry*.

[2] Jackson RJ, Hellen CUT, Pestova TV (2010). The mechanism of eukaryotic translation initiation and principles of its regulation. *Nature Reviews Molecular Cell Biology*.

[3] Wells SE, Hillner PE, Vale RD, Sachs AB (1998). Circularization of mRNA by eukaryotic translation initiation factors. *Molecular Cell*.

[4] Yanagiya A, Svitkin YV, Shibata S, Mikami S, Imataka H, Sonenberg N (2009). Requirement of RNA binding of mammalian eukaryotic translation initiation factor 4GI (eIF4GI) for efficient interaction of eIF4E with the mRNA cap. *Molecular and Cellular Biology*.

[5] Oberer M, Marintchev A, Wagner G (2005). Structural basis for the enhancement of eIF4A helicase activity by eIF4G. *Genes and Development*.

[6] Ozes AR, Feoktistova K, Avanzino BC (2011). Duplex unwinding and ATPase activities of the DEAD-box helicase eIF4A are coupled by eIF4G and eIF4B. *Journal of Molecular Biology*.

[7] Lefebvre AK, Korneeva NL, Trutschl M (2006). Translation initiation factor eIF4G-1 binds to eIF3 through the eIF3e subunit. *Journal of Biological Chemistry*.

[8] He H, Von der Haar T, Singh CR (2003). The yeast eukaryotic initiation factor 4G (eIF4G) HEAT domain interacts with eIF1 and eIF5 and is involved in stringent AUG selection. *Molecular and Cellular Biology*.

[9] Sonenberg N, Hinnebusch AG (2009). Regulation of translation initiation in eukaryotes: mechanisms and biological targets. *Cell*.

[10] Browning KS (2004). Plant translation initiation factors: it is not easy to be green. *Biochemical Society Transactions*.

[11] Browning KS (1996). The plant translational apparatus. *Plant Molecular Biology*.

[12] Mayberry LK, Leah Allen M, Dennis MD, Browning KS (2009). Evidence for variation in the optimal translation initiation complex: plant eIF4B, eIF4F, and eIF(iso)4F differentially promote translation of mRNAs. *Plant Physiology*.

[13] Mayberry LK, Allen ML, Nitka KR (2011). Plant cap-binding complexes eukaryotic initiation factors eIF4F and eIFISO4F: molecular specificity of subunit binding. *Journal of Biological Chemistry*.

[14] Altschul SF, Wootton JC, Zaslavsky E, Yu YK (2010). The construction and use of log-odds substitution scores for multiple sequence alignment. *PLoS Computational Biology*.

[15] Grigoriev IV, Nordberg H, Shabalov I (2012). The genome portal of the department of energy joint genome institute. *Nucleic Acids Research*.

[16] Goodstein DM, Shu S, Howson R (2012). Phytozome: a comparative platform for green plant genomics. *Nucleic Acids Research*.

[17] Bombarely A, Menda N, Tecle IY (2011). The Sol Genomics Network (solgenomics.net): growing tomatoes using Perl. *Nucleic Acids Research*.

[18] Shulaev V, Sargent DJ, Crowhurst RN (2010). The genome of woodland strawberry (*Fragaria vesca*). *Nature Genetics*.

[19] Gasteiger E, Gattiker A, Hoogland C, Ivanyi I, Appel RD, Bairoch A (2003). ExPASy: the proteomics server for in-depth protein knowledge and analysis. *Nucleic Acids Research*.

[20] Larkin MA, Blackshields G, Brown NP (2007). Clustal W and clustal X version 2.0. *Bioinformatics*.

[21] Katoh K, Toh H (2008). Recent developments in the MAFFT multiple sequence alignment program. *Briefings in Bioinformatics*.

[22] Ruud KA, Kuhlow C, Goss DJ, Browning KS (1998). Identification and characterization of a novel cap-binding protein from *Arabidopsis thaliana*. *Journal of Biological Chemistry*.

[23] Moore MJ, Bell CD, Soltis PS, Soltis DE (2007). Using plastid genome-scale data to resolve enigmatic relationships among basal angiosperms. *Proceedings of the National Academy of Sciences of the United States of America*.

[24] Marintchev A, Edmonds KA, Marintcheva B (2009). Topology and regulation of the human eIF4A/4G/4H helicase complex in translation initiation. *Cell*.

[25] Marintchev A, Wagner G (2005). eIF4G and CBP80 share a common origin and similar domain organization: implications for the structure and function of eIF4G. *Biochemistry*.

[26] Gallie DR, Browning KS (2001). eIF4G functionally differs from eIFiso4G in promoting internal initiation, cap-independent translation, and translation of structured mRNAs. *Journal of Biological Chemistry*.

[27] Robaglia C, Caranta C (2006). Translation initiation factors: a weak link in plant RNA virus infection. *Trends in Plant Science*.

[28] Lellis AD, Allen ML, Aertker AW (2010). Deletion of the eIFiso4G subunit of the *Arabidopsis* eIFiso4F translation initiation complex impairs health and viability. *Plant Molecular Biology*.

[29] Yoshii M, Nishikiori M, Tomita K (2004). The *Arabidopsis* cucumovirus multiplication 1 and 2 loci encode translation initiation factors 4E and 4G. *Journal of Virology*.

[30] Metz AM, Browning KS (1996). Mutational analysis of the functional domains of the large subunit of the isozyme form of wheat initiation factor eIF4F. *Journal of Biological Chemistry*.

[31] Cavalier-Smith T (2010). Kingdoms Protozoa and Chromista and the eozoan root of the eukaryotic tree. *Biology Letters*.

[32] Nicaise V, Gallois JL, Chafiai F (2007). Coordinated and selective recruitment of eIF4E and eIF4G factors for potyvirus infection in *Arabidopsis thaliana*. *The FEBS Letters*.

[33] Cheng S, Gallie DR (2010). Competitive and noncompetitive binding of eIF4B, eIF4A, and the poly(A) binding protein to wheat translation initiation factor eIFiso4G. *Biochemistry*.

[34] Lax S, Fritz W, Browning K, Ravel J (1985). Isolation and characterization of factors from wheat germ that exhibit eukaryotic initiation factor 4B activity and overcome 7-methylguanosine 5’-triphosphate inhibition of polypeptide synthesis. *Proceedings of the National Academy of Sciences of the United States of America*.

