# Supplementary material for: The eIF4F and eIFiso4F Complexes of Plants: An Evolutionary Perspective
Source: Comp Funct Genomics. 2012 May 7;2012:287814. doi: 10.1155/2012/287814 (PMC3352336; doi:10.1155/2012/287814)
Supplement: Supplementary file 1 — eIF4G and eIFiso4G gene names used for alignments in this study. Gene ID refers to identifiers used for a gene locus or transcript in the NCBI database or the genome project for that organism. [file 287814.f1.pdf]

Supplementary Table 1. eIF4G and eIFiso4G gene names used in alignments and gene project or NCBI locus/transcript ID. Expressed sequence tag (EST) sourced sequences are italicized.

Plant eIF4G	
Gene	ID
Adiantum.capillus-veneris.4G	*BP915997.1*
Amborella.trichopoda.4G	*CV011294.1*
Aquilegia.coerulea.4G	AcoGoldSmith_v1.000275m
Arabidopsis.thaliana.4G	AT3G60240
Arabidosis.lyrata.4G	939058
Brachypodium.distachyon.4G	Bradi1g25000
Brachypodium.distachyon.4G2	Bradi5g17250.1
Carica.papaya.4G	evm.TU.supercontig_52.78
Ceratopteris.richardii.4G	*CV011294.1*
Chlamydomonas.reinhardtii.4G	Cre17.g696250
Citrus.clementina.4G	clementine0.9_000112m.g
Cucumis.sativus.4G1	Cucsa.265850
Cucumis.sativus.4G2	Cucsa.256840.1
Eucalyptus.grandis.4G	Eucgr.E01663
Fragaria.vesca.4G	gene03047-v1.0-hybrid
Glycine.max.4G1	Glyma07g13380
Glycine.max.4G2	Glyma03g25390
Glycine.max.4G3	Glyma08g45420
Glycine.max.4G4	Glyma01g23500
Manihot.esculenta.4G1	cassava4.1_000086m.g
Manihot.esculenta.4G2	cassava4.1_000081m.g
Medicago.truncatula.4G	Medtr8g008440
Micromonas.CCMP1545.4G	MICPUCDRAFT_6369, MICPUCDRAFT_64212
Micromonas.RCC299.4G	MICPUN_71782
Mimulus.guttatus.4G1	mgv1a000123m.g
Mimulus.guttatus.4G2	mgv1a000230m.g
Oryza.sativa.4G	LOC_Os07g36940
Ostreococcus.lucimarinus.4G	OSTLU_7162 (and upstream)
Ostreococcus.tauri.4G	Ot03g00710 (and upstream)
Physcomitrella.patens.4G1	Pp1s96_61V6
Physcomitrella.patens.4G2	Pp1s96_133V6
Picea.glauca.4G	*BT108681.1, BT111027.1*
Picea.sitchensis.4G	*DR535248.1*

Plant eIFiso4G	
Gene	ID
Aquilegia.coerulea.iso4G1	AcoGoldSmith_v1.001135m
Arabidopsis.lyrata.iso4G1	495887
Arabidopsis.lyrata.iso4G2	481263
Arabidopsis.lyrata.iso4G3	353746
Arabidopsis.thaliana.iso4G1	AT5G57870.1
Arabidopsis.thaliana.iso4G2	AT2G24050.1
Aristolochia.fimbriata.iso4G	*FD763516.1*
Brachypodium.distachyon.iso4G	Bradi5g14690.1
Carica.papaya.iso4G	evm.model.supercontig_46.175
Chlamydomonas.reinhardtii.iso4G	CHLREDRAFT_147254
Chlorella.variabilis.iso4G	CHLNCDRAFT_142387
Citrus.clementina.iso4G1	clementine0.9_002686m
Citrus.clementina.iso4G2	clementine0.9_031294m
Citrus.sinensis.iso4G1	orange1.1g045399m
Citrus.sinensis.iso4G2	orange1.1g003543m
Cucumis.sativus.iso4G1	Cucsa.170670.1
Cucumis.sativus.iso4G2	Cucsa.271430.1
Eucalyptus.grandis.iso4G1	Eucgr.C00248.1
Eucalyptus.grandis.iso4G2	Eucgr.C00247.1
Fragaria.vesca.iso4G1	gene15000-v1.0-hybrid
Fragaria.vesca.iso4G2	gene10104-v1.0-hybrid
Glycine.max.iso4G1	Glyma17g08030.1
Glycine.max.iso4G2	Glyma06g30880.1
Glycine.max.iso4G3	Glyma02g36680.1
Glycine.max.iso4G4	Glyma04g23560.1
Manihot.esculenta.iso4G1	cassava4.1_001915m
Manihot.esculenta.iso4G2	cassava4.1_001934m
Medicago.truncatula.iso4G	Medtr8g145060.1
Micromonas.CCMP1545.iso4G	MICPUCDRAFT_42607
Micromonas.RCC299.iso4G	fgenesh2_pg.C_Chr_10000089
Mimulus.guttatus.iso4G1	mgv1a001641m
Mimulus.guttatus.iso4G2	mgv1a002526m
Oryza.sativa.iso4G1	LOC_Os04g42140.1

Heterokont eIF4G	
Gene	ID
Albugo.laibachii.4G1	CCA22694.1
Albugo.laibachii.4G2	CCA13992.1
Aureococcus.anophagefferens.4G	AURANDRAFT_21900
Cyanidioschyzon.merolae.4G1	CMS073C
Cyanidioschyzon.merolae.4G2	CMQ348C
Ectocarpus.siliculosus.4G1	CBN76090.1
Ectocarpus.siliculosus.4G2	CBJ33927.1
Phaeodactylum.tricornutum.4G1	PHATRDRAFT_47943
Phaeodactylum.tricornutum.4G2	PHATRDRAFT_44298
Phytophthora.infestans.4G1	PITG_15393
Phytophthora.infestans.4G2	PITG_14278
Thalassiosira.pseudonana.4G1	THAPSDRAFT_264322 (and upstream)
Thalassiosira.pseudonana.4G2	THAPSDRAFT_261777

Plant eIF4G	
Gene	ID
Populus.trichocarpa.4G1	POPTR_0014s05150
Populus.trichocarpa.4G2	POPTR_0002s14110
Prunus.persica.4G	ppa000085m.g
Ricinus.communis.4G	29709.t000028
Selaginella.moellendorffii.4G	104901 (and upstream)
Setaria.italica.4G1	Si028648m.g
Setaria.italica.4G2	Si012624m.g
Solanum.lycopersicum.4G1	Solyc01g088700.2.1
Solanum.lycopersicum.4G2	Solyc08g077700.2.1
Solanum.tuberosum.4G1	PGSC0003DMP400003096
Solanum.tuberosum.4G2	PGSC0003DMP400003312
Sorghum.bicolor.4G1	Sb02g036110.1
Sorghum.bicolor.4G2	Sb06g024070.1
Thellungiella.halophila.4G	Thhalv10005736m.g
Theobroma.cacao.4G	CGD0004103
Triticum.aestivum.4G	AEQ49596.1
Vitis.vinifera.4G	LOC100261523
Volvox.carteri.4G	VOLCADRAFT_8041 (and upstream)
Zea.mays.4G	GRMZM2G386430
Zea.mays.4G2	GRMZM2G153162_T01
Zea.mays.4G3	GRMZM5G821988_T01

Plant eIFiso4G	
Gene	ID
Oryza.sativa.iso4G2	LOC_Os02g39840.1
Ostreococcus.lucimarinus.iso4G	fgenesh1_pg.C_Chr_19000003
Physcomitrella.patens.iso4G1	Pp1s65_89V6.1
Physcomitrella.patens.iso4G2	Pp1s65_80V6.1
Physcomitrella.patens.iso4G3	Pp1s65_106V6.1
Physcomitrella.patens.iso4G4	Pp1s34_174V6.1
Physcomitrella.patens.iso4G5	Pp1s34_182V6.1
Pinus.radiata.iso4G	*GO096175.1*
Pinus.taeda.iso4G	*DN455542.1*
Populus.trichocarpa.iso4G1	POPTR_0018s11310.1
Populus.trichocarpa.iso4G2	POPTR_0018s02700.1
Populus.trichocarpa.iso4G3	POPTR_0006s19600.1
Populus.trichocarpa.iso4G4	POPTR_0006s28110.1
Prunus.persica.iso4G1	ppa001769m
Prunus.persica.iso4G2	ppa001554m
Ricinus.communis.iso4G	27455.m000039
Selaginella.moellendorffii.iso4G	437322
Setaria.italica.iso4G1	Si009406m
Setaria.italica.iso4G2	Si016412m
Solanum.lycopersicum.iso4G1	Solyc12g009960.1.1
Solanum.lycopersicum.iso4G2	Solyc07g005810.2.1
Solanum.tuberosum.iso4G1	PGSC0003DMP400013944
Solanum.tuberosum.iso4G2	PGSC0003DMP400019801
Sorghum.bicolor.iso4G	Sb06g021600.1
Thellungiella.halophila.iso4G1	Thhalv10012738m
Thellungiella.halophila.iso4G2	Thhalv10000058m
Theobroma.cacao.iso4G1	CGD0032058
Theobroma.cacao.iso4G2	CGD0031876
Triticum.aestivum.iso4G	AAA16209.1
Vitis.vinifera.iso4G1	GSVIVT01035980001
Vitis.vinifera.iso4G2	GSVIVT01023638001
Volvox.carteri.iso4G	VOLCADRAFT_103732
Zea.mays.iso4G1	GRMZM2G157061_T01
Zea.mays.iso4G2	GRMZM2G098577_T01
